# Electron Transfer Flavoprotein (ETF) *α* Controls Blood Vessel Development by Regulating Endothelial Mitochondrial Bioenergetics and Oxygen Consumption

**DOI:** 10.1155/2022/7969916

**Published:** 2022-03-11

**Authors:** Yi Yan, Yingyi Xu, Xuewen Yang, Zhonghao Li, Kaiyuan Niu, Chenxin Liu, Ming Zhao, Qingzhong Xiao, Wei Wu

**Affiliations:** ^1^Department of Cardiology, Translational Research Center for Regenerative Medicine and 3D Printing Technologies, The Third Affiliated Hospital of Guangzhou Medical University, Guangzhou 510150, China; ^2^State Key Laboratory of Organ Failure Research, Southern Medical University, Guangzhou 510000, China; ^3^Department of Pathophysiology, Key Lab for Shock and Microcirculation Research of Guangdong Province, School of Basic Medical Sciences, Southern Medical University, Guangzhou 510515, China; ^4^William Harvey Research Institute, Barts and The London School of Medicine and Dentistry, Queen Mary University of London, London, EC1M 6BQ, UK; ^5^Key Laboratory of Cardiovascular Diseases at The Second Affiliated Hospital and Guangzhou Municipal and Guangdong Provincial Key Laboratory of Protein Modification and Degradation, School of Basic Medical Sciences, Guangzhou Medical University, Xinzao Town, Panyu District, Guangzhou, Guangdong 511436, China

## Abstract

While impairment of vascular homeostasis induced by hypercholesterolemia is the first step of cardiovascular diseases, the molecular mechanism behind such impairment is not well known. Here, we reported that high-cholesterol diet (HCD) induced defective vessel sprouting in zebrafish larvae. Electron transfer flavoprotein subunit *α* (ETF*α*) (encoded by the ETFA gene), a protein that mediates transfer of electrons from a series of mitochondrial flavoenzymes to the respiratory chain, was downregulated in HCD-fed zebrafish and in endothelial cells treated with oxidized low-density lipoprotein. Knockdown of ETF*α* with morpholino antisense oligonucleotides reproduced vascular sprouting defects in zebrafish larvae, while replenishing with exogeneous ETFA mRNA could successfully rescue these defects. ETFA knockdown in endothelial cells reduces cell migration, proliferation, and tube formation in vitro. Finally, knockdown of ETFA in endothelial cells also reduced fatty acid oxidation, oxygen consumption rate, and hypoxia-inducible factor-1*α* (HIF1*α*) protein levels. Taken together, we demonstrate that downregulation of ETF*α* is involved in hypercholesterolemia-induced defective vessel sprouting in zebrafish larvae via inhibition of endothelial proliferation and migration. The molecular mechanism behind this phenomenon is the decrease of HIF1*α* induced by downregulation of ETF*α* in endothelial cells. This work suggests that disturbance of ETF*α*-mediated oxygen homeostasis is one of the mechanisms behind hypercholesterolemia-induced vascular dysfunction.

## 1. Introduction

Hypercholesterolemia has been linked to cardiovascular disease (CVD) for decades, which could lead to premature atherosclerosis-based cardiovascular diseases and death, but the underlying mechanism is still far away from elucidation [1, 2]. In our previous work, we utilized high-cholesterol diet- (HCD-) fed zebrafish larvae to mimic hypercholesterolemia and preatherosclerosis to investigate the molecular mechanism between hypercholesterolemia and atherosclerosis [3, 4]. The zebrafish larvae fed a HCD acquired a broad spectrum of phenotypes which include not only hypercholesterolemia and atherosclerotic plaques like lesions but also delayed development and vascular deformities at 15 days postfertilization (dpf). Importantly, the severity of deformities like vascular defects was positively correlated with the increase of cholesterol concentration in diet, which suggests that hypercholesterolemia compromises the vessel development. Endothelial cells play an important role in the formation of vessel under both physiological and pathological conditions [5]. As damage of the endothelium is considered as the initiation step of atherosclerosis, it is necessary to investigate the relationship between hypercholesterolemia and vascular deformities in zebrafish. iTRAQ analysis of normal- and HCD-fed zebrafish larvae found a total of 63 differentially expressed proteins [3]. More than 12.2% of those 63 candidates were metabolic activities related, and 14.3% of them were mitochondrion located, which suggest that mitochondria are probably related to hypercholesterolemia and deformities. However, the differentially expressed mitochondrion elements is a driving force of those phenotypes or just a subsequent bystander adaptation needs further investigation. Although the volume of mitochondria in endothelial cells is not abundant as some cell types like hepatocytes, the importance of mitochondria in EC homeostasis is receiving more and more attention [6–9]. Recently, there are many studies that revealed that endothelial metabolism is a key driver of vessel sprouting in addition to well-known growth factor-based signaling [8, 9]. Endothelial mitochondria are found not only as a supplier of energy but also as a signal station getting involved in many biological activities like angiogenesis [10, 11]. To reveal the relationship between mitochondrion function and hypercholesterolemia-related vascular deformities, we pick electron transfer flavoprotein-*α* (ETF*α*), one of the differentially expressed mitochondrial proteins in iTRAQ analysis, to carry out our investigation. ETF*α* is an *α* subunit of dimeric enzyme which locates in the mitochondrial matrix space and collects reducing power from multiple metabolic pathways, funneling electrons into the respiratory chain [12]. Its central position in mitochondrial bioenergetics enables ETF*α* an appropriate candidate to evaluate the role of mitochondria in vascular development and homeostasis. Loss of function in ETF could induce severe symptoms, the so-called multiple acyl-CoA dehydrogenase deficiency (MADD), in human beings [12–15]. Many patients with ETF deficiency are neonatal lethal and only a fraction of patients with milder signs survives to adulthood [12–15]. The severity of the symptoms induced by ETF deficiencies depends on the residual enzyme activity [12, 16]. To mimic the milder downregulation of ETF*α* induced by hypercholesterolemia but not severely demolished like MADD, we use morpholino to knockdown ETFA in zebrafish embryos to leave residual ETFA function. Injection of ETFA morpholino led to less than 42% mortality at 48 hpf in zebrafish larvae, and the surviving larvae had a broad spectrum of deformities, which include vascular malformation. Knockdown of ETF*α* in endothelial cells with shRNA could reduce cell migration, proliferation, and tube formation in vitro. The fatty acid oxidation (FAO), oxygen consumption rate, and HIF1*α* protein expression were also impaired by ETFA knockdown. Our work provides first-hand evidence to support a critical role for ETF*α* or mitochondria in vessel sprouting and development. This work may shed light on the underlying link between hypercholesterolemia and CVD.

## 2. Materials and Methods

### 2.1. Culture and Maintenance of Zebrafish Larvae

The adult zebrafish (*Danio rerio*) were maintained with an automatic fish housing system at 28°C following standard protocols. Transgenic lines Tg (Flk: eGFP) were obtained from Professor Yiyue Zhang at South China University of Technology. Zebrafish embryos and larvae were raised in 10% egg water under a 14 h-10 h light-dark cycle, and 0.003% 1-phenyl-2-thiourea (PTU) (Sigma, P7629) was added to the egg water since 24 hpf to prevent pigment formation.

### 2.2. iTRAQ Data Analysis

The procedure of isobaric tags for relative and absolute quantitation (iTRAQ) is described previously. The MS/MS raw data were blasted against the Ensembl Danio Rerio database for peptide identification and quantification using Mascot 2.3.02. Proteins with FDR ≤ 0.01 were qualified for further quantification. A fold change cutoff ratio (FC) of ≥1.2 or ≤0.833 was set as the threshold to identify differently expressed proteins. To calculate the fold change between sample *A* and sample *B*, the formula is as follows: FC, *k* = *RAk*/*RBk* (*R* denotes the relative quantitative value of the protein, *k* denotes the protein, *A* and *B* denote different groups, and *RAk* and *RBk* denote the relative quantitative values of protein *k* in groups *A* and *B*. Student's *t*-test was performed with the relative quantitative value of the peptide on each protein to judge the significance of differentially expressed proteins between groups, and the default *P* value was <0.05. To make the data obey the normal distribution, the relative quantitative value of peptides needs log2 transformation. The calculation formula is as follows: *Pk* = *t*.test(log2(*UAj*), log2(*UBj*), *j* ∈ *k*) (*U* denotes the relative quantitative value of the peptide; *j* denotes the peptide). The differentially expressed proteins were annotated with Gene Ontology (GO) annotation. QPCR and Western blot were carried out to confirm the data from iTRAQ analysis.

### 2.3. Morpholino and mRNA Injection

Morpholinos (MOs) were purchased from GeneTools (Philomath, OR, USA) and dissolved in DEPC water. Morpholinos were designed to prevent ETFA splicing (ETFA MO) by targeting the exon 3-intron 2 junction. Zebrafish embryos were microinjected with 5 ng ETFA or control MO at a one-cell stage. The respective MO sequences are ETFA MO: 5′-GCTCTGCAACCTGTGCAAAATGAGA-3′; control MO: 5′-CCTCTTACCTCAGTTACAATTTATA-3′. The overexpression ETFA-mRNA was obtained from the in vitro transcription of ABSR-RNA by using the mMESSAGE mMACHINE™ SP6 Transcription Kit (Invitrogen™, AM1340). PCS2+ vector (Addgene, MA, USA) was used to construct a plasmid with the ETFA-CDS sequence, and ETFA-mRNA was obtained by HpaI digestion, followed by in vitro transcription. We injected 50 pg, 100 pg, 200 pg, and 500 pg mRNA or/and 5 ng MO at the one-cell embryo stage.

### 2.4. RNA Preparation and PCR

Total RNAs of zebrafish embryos were extracted from MO-injected embryo by using TRIzol reagent according to the manufacturer's instructions (Invitrogen, 15596018). The extracted total RNA was used to generate the first-strand cDNA (1 *μ*g) by using HiScript II Reverse Transcriptase (Vazyme, R201) with a random primer. The primers used for PCR are as follows: ETFA forward: 5′-ACATTGAACGCCATCACCGC-3′; ETFA reverse: 5′-CGCCGGCTCGAATGAAGTTC-3′.

### 2.5. In Vivo Confocal Microscopy

For in vivo confocal microscopy, zebrafish larvae were anesthetized with 0.02% tricaine and mounted in 0.5% low-melting agarose and observed with an Olympus FV1000 confocal laser scanning microscope (Olympus, Tokyo, Japan). Images of zebrafish larvae were captured every 200 nm and analyzed using the Olympus FluoView software. The Z-step of imaging was 10 *μ*m. Quantitative analysis of the ISVs and its morphology was conducted following the processes described in reference [17].

### 2.6. Cell Culture

The primary human umbilical vein endothelial cell (HUVECs) was purchased from ATCC, USA. HUVECs were maintained in Dulbecco's modification of Eagle's medium (DMEM)/F12 (Life Technologies) supplemented with 10% (*v*/*v*) fetal bovine serum (FBS) (Life Technologies), 2 mM L-glutamine (Life Technologies), 0.1 mg ml-1 heparin (Sigma-Aldrich), 0.05 mg ml-1 endothelial cell growth supplement (Sigma-Aldrich), 100 U/ml penicillin, and 100 mg/ml streptomycin and used between passages 4 and 9.

### 2.7. Lentivirus Transfection

HUVECs were seeded in 6-well plates overnight with a density of 1 × 10^5 and infected with control or gene-specific lentivirus (MOI: 10). At 18–24 h postinfection, the infection medium was replaced with DMEM containing 10% FBS and cells were cultured for further 24–48 hours.

### 2.8. Tube Formation Assay

Matrigel (BD Bioscience) (150 *μ*l) was added to the 48-well plate precooled at 4°C and incubated at 37°C for 2 h in a 5% CO_2_ incubator. Cells were dissociated into single cells and added onto the solidified Matrigel at a density of 2.5 × 10^4, followed by an incubation at 37°C for 3–6 h. The tube formation was observed by a microscope. Networks of tube-like structures were measured using ImageJ software.

### 2.9. Wound Healing Assay

After planting the virus-treated cells in 6-well plates and growing to 95% confluence, a neat line was drawn in each well using a sterile 200 *μ*l pipette tip. Cells were washed twice with PBS and immediately photographed as “S0” after adding fresh culture medium in the absence or presence of 2 *μ*M mitomycin C (2 *μ*M). Six–twelve hours later, cells were photographed again and used as “Sx.” NIH ImageJ software was used to measure the wound healing area (the gap area at S0 minus the gap area at Sx).

### 2.10. Fatty Acid Oxidation Assay

Oxidation of exogenous and endogenous fatty acids was measured using the XF Palmitate-BSA FAO Substrate with the XF Cell Mito Stress Test. Cells were seeded at 40,000 cells per well on Seahorse XF96 cell culture plates (Seahorse Bioscience Europe, Copenhagen, Denmark). The measurement of oxygen consumption was performed at 10 min intervals (2 min mixing, 2 min recovery, and 6 min measuring) for 3 h using the Seahorse XF96 analyzer.

### 2.11. Protein Extraction

1 to 3 dpf zebrafish embryos were collected by breaking their membranes and adding 0.3 mM PMSF to remove yolk sac, followed by centrifugation at 200 × g at 4°C for 5 min. The treated embryos or cells were washed with PBS, and proteins were extracted by using RIPA lysis buffer (Beyotime, China), with protease and phosphatase inhibitors. Following centrifugation of the lysates at 13,000 × g at 4°C for 10 min, the supernatants were collected and protein concentrations were quantified using a BCA assay kit (Beyotime, China).

### 2.12. Western Blot

Equal amounts of protein from each sample were separated by 10% SDS-PAGE and transferred to PVDF membrane which were blocked with 5% skimmed milk (Sigma-Aldrich; Merck KGaA) in TBS solution with 0.1% Tween-20 (Sangon Biotech Co. Ltd.) at room temperature for 1 h and then incubated with the primary antibodies (1 : 1000) at 4°C overnight with rocking. The membrane was washed with TBST for 5 min 3 times and incubated with the secondary antibody (1 : 5000) at room temperature for 1 h. The reactive bands were detected and observed via an enhanced chemiluminescence (ECL) kit. Densitometric quantifications of bands were done with ImageJ software (National Institutes of Health) using GAPDH or *β*-actin as an internal reference.

### 2.13. Statistical Analysis

Data in graphs are presented as means ± SE. Differences among experimental groups were evaluated by one-way ANOVA. *P* < 0.05 was considered statistically significant. ^∗^*P* < 0.01, ^∗∗^*P* < 0.001, ^∗∗∗^*P* < 0.0001, and ^∗∗∗∗^*P* < 0.00001.

## 3. Results

### 3.1. High-Cholesterol Diet Impairs Vessel Development in Zebrafish Larvae

**Figure 1:**
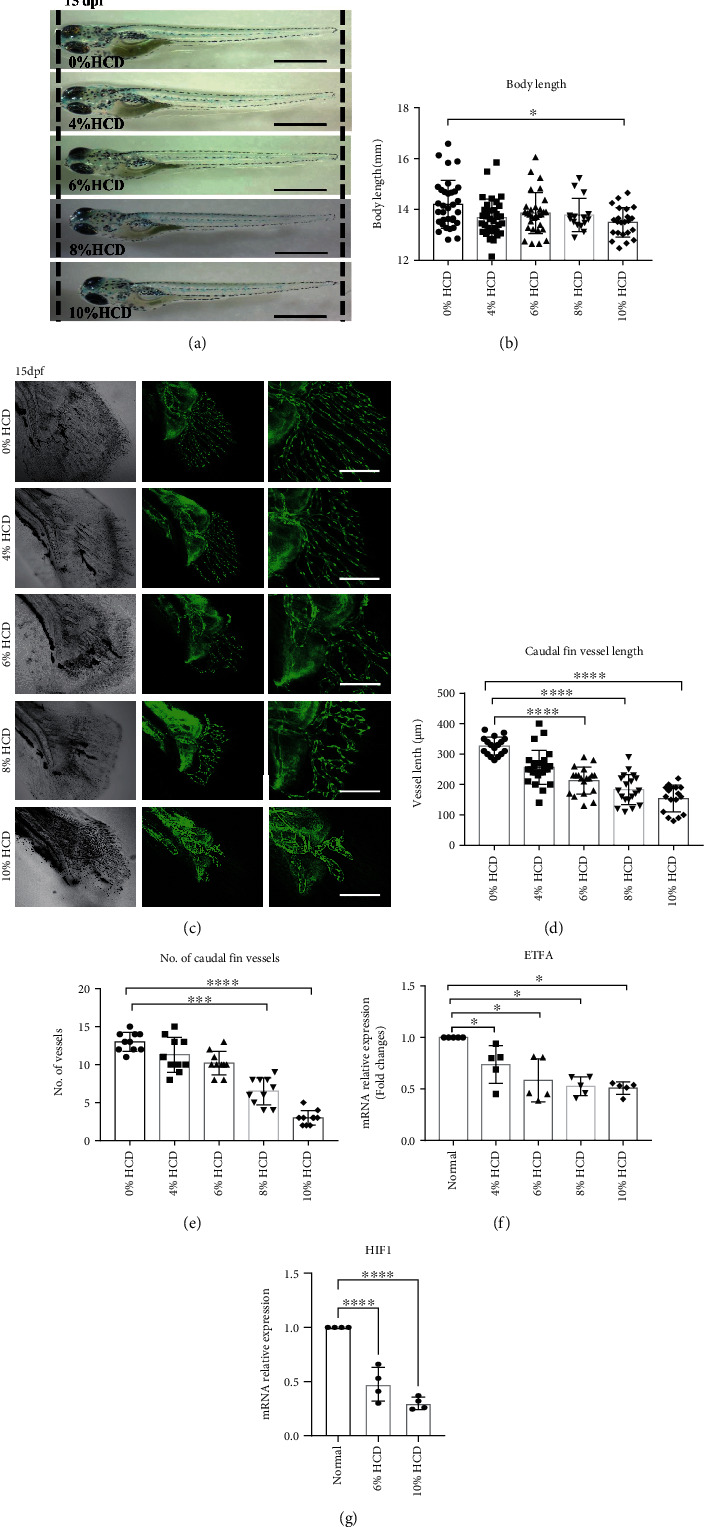
High-cholesterol diet-induced vascular defects and decreased ETFA expression in zebrafish larvae. *Tg Flk1: EGFP* zebrafish larvae were fed with different concentrations of high-cholesterol-containing diet (HCD) from 5 days postfertilization (dpf) to 15 dpf, followed by various evaluations. (a, b) HCD induced shorten body length of zebrafish embryos. (a) Representative phage-contrast images of 15dpf larvae fed with different concentrations of HCD (0%, 4%, 6%, 8%, and 10%), scale bar = 3 mm; (b) statistical analysis of the total body length (mm) of the zebrafish larvae (one-way ANNOVA, ^∗^*P* < 0.01, *n* = 30); (c–e) HCD-induced developmental vascular defects in zebrafish larvae. (c) Representative image of vessel morphologies in caudal fins of 15dpf *Tg Flk1: EGFP* zebrafish larvae. Green EGFP signals represent VEGR2^+^ vessels, scale bar = 200 *μ*m. (d) Statistical analysis of vessel length (*μ*m) in caudal fins of HCD-fed zebrafish larvae (one-way ANNOVA, ^∗∗∗∗^*P* < 0.00001, *n* = 14); (e) statistical analysis of the vessel number in caudal fins of HCD-fed zebrafish larvae (one-way ANNOVA, ^∗∗∗^*P* < 0.0001, ^∗∗∗∗^*P* < 0.00001, *n* = 10); (f) RT-qPCR analysis of ETFA gene expression in HCD-fed zebrafish larvae. ^∗^*P* < 0.01 (one-way ANNOVA, *n* = 5); (g) RT-qPCR analysis of HIF1 gene expression in HCD-fed zebrafish larvae. ^∗∗∗∗^*P* < 0.00001 (one-way ANNOVA, *n* = 4).

Feeding a high-cholesterol diet (HCD) to *Tg Flk1: eGFP* zebrafish larvae for 10 days was used to mimic atherosclerosis-like phenotypes in vivo, as previously reported [3]. Expectedly, it was found that HCD contained different concentrations of cholesterol which caused a trend of developmental delay, which is characterized with decreased body length and with significant shorter body length in zebrafish larvae fed a 10% cholesterol diet (Figures 1(a) and 1(b)). Importantly, we observed that both the number and length of the blood vessels in caudal fins were significantly decreased by HCD feeding in a dose-dependent manner (Figures 1(c)–1(e)). And the angiogenesis-related gene HIF1 was also decreased in HCD-fed larvae (Figure 1(g)).

### 3.2. High-Cholesterol Diet Reduces the ETF*α* Protein Level in Zebrafish

In order to reveal the differences of protein profiles between normal and atherosclerotic zebrafish larvae (diet fed containing 4% cholesterol), the tissues of the two groups mentioned above were subjected to iTRAQ analysis, as described in our previous study [3]. After reanalyzing of the iTRAQ data, it was found that several mitochondrion-related proteins were differentially expressed (Table 1). ETF*α* is one of those mitochondrion-related proteins. Data from RT-qPCR (Figure 1(f) and Figure S2) revealed that ETF*α* is the only one that downregulated in 4% to 10% HCD-fed zebrafish larvae and consistent with the result found by iTRAQ. So ETF*α* was selected to investigate its role in hypercholesterolemia-induced defective vessel growth in this work.

### 3.3. Knockdown of ETF*α* Caused Vessel Developmental Defects in Zebrafish Larvae and Such Defects Were Rescued by Exogenous Zebrafish ETFA mRNA

**Figure 2:**
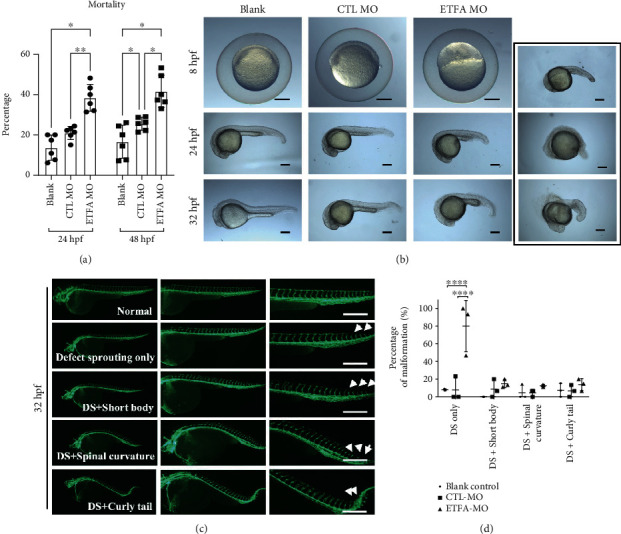
ETF*α* knockdown caused developmental delay and deformities. *Tg Flk1: EGFP* zebrafish embryos were injected with 5 ng blank control, control morpholino (Ctl-MO), or *ETFA* target morpholino (*ETFA*-MO) and were inspected at 8, 24, 32, or 48 hpf, respectively. (a) The mortality was calculated and compared among blank, control MO-treated, and ETFA MO-treated groups (one-way ANNOVA, ^∗^*P* < 0.01, *n* = 5); (b) developmental status of embryos injected with blank, control MO, and ETFA MO was observed at 8, 24, and 32 hpf, respectively, under a bright field. The photographs in the black box exhibit different deformities of ETFA MO-treated embryos; scale bar = 200 *μ*m; (c) developmental defects were observed at 32 hpf under a confocal microscope. Data presented in (c) were representative fluorescent images of malformation in ETFA-MO-injected embryos at 32 hpf, which include defect sprouting (DS) alone, DS with a short body, DS with spinal curvature, and DS with a curly tail; scale bar = 100 *μ*m; (d) quantitative data of the percentage of malformation in different groups (one-way ANNOVA, ^∗∗∗∗^*P* < 0.00001, *n* = 3).

**Figure 3:**
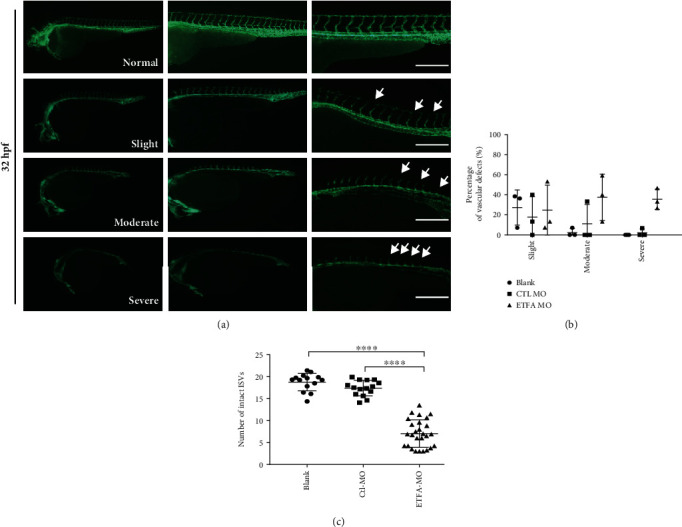
ETF*α* knockdown caused defective vascular sprouting at 32 hpf. (a) Representative fluorescent images showing different types of vascular defects in *ETFA*-MO-injected *Tg Flk1: EGFP* embryos at 32 hpf, including slight (incomplete DLAVs), moderate (short ISVs), and severe (inadequate sprouting); scale bar = 100 *μ*m; (b) quantitative data of the different vascular defects at 32 hpf (one-way ANNOVA, *n* = 3); (c) the total numbers of intact intersegmental vessels (ISVs) (one-way ANNOVA, ^∗∗∗∗^*P* < 0.00001, *n* = 15).

**Figure 4:**
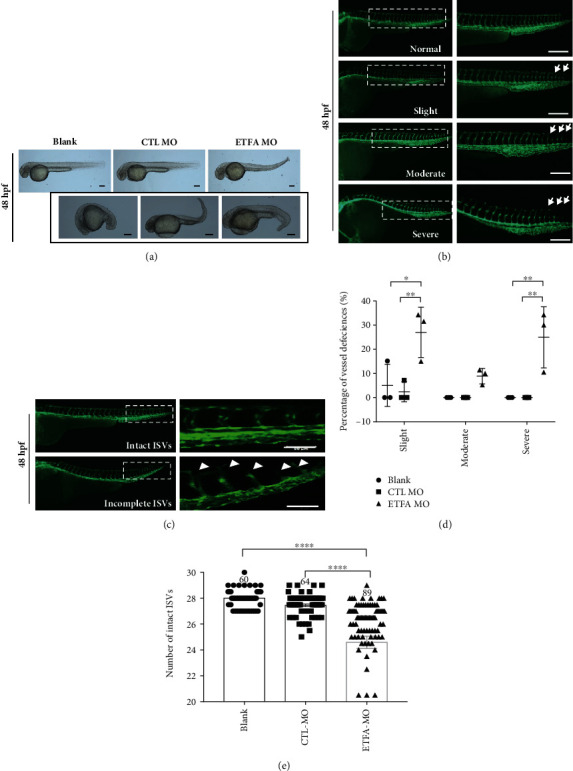
ETF*α* knockdown induced deformities at 48 hpf. (a) Representative bright field images showing different types of deformities in *ETFA*-MO-injected *Tg Flk1: EGFP* embryos at 48 hpf. The photographs in the black box exhibit different deformities of ETFA MO-treated larvae; scale bar = 200 *μ*m; (b) representative fluorescent images showing different types of vascular defects in *ETFA*-MO-injected *Tg Flk1: EGFP* embryos at 32 hpf. These defects were classified as slight (intact ISVs less than 27 but more than 25), moderate (intact ISVs less than 25 but more than 20), and severe defect (intact ISVs less than 20). The white arrows indicate where the vessel is defective. DLAV: dorsal longitudinal anastomotic vessel; ISVs: intersegmental vessels; DA: dorsal aorta, scale bar = 100 *μ*m; (c) ETF*α* inhibition impaired segmental vessel sprouting and their fusion with DLAV in *Tg Flk1: EGFP* zebrafish larvae at 48 hpf. Data presented here were representative fluorescent images of vascular sprouting defects of segmental vessels; (d) quantitative data of the different vascular defects at 48 hpf (one-way ANNOVA, *n* = 3); (e) quantitative data of intact ISVs in zebrafish larvae at 48 hpf (one-way ANNOVA, ^∗∗∗∗^*P* < 0.00001, *n* = 60).

**Figure 5:**
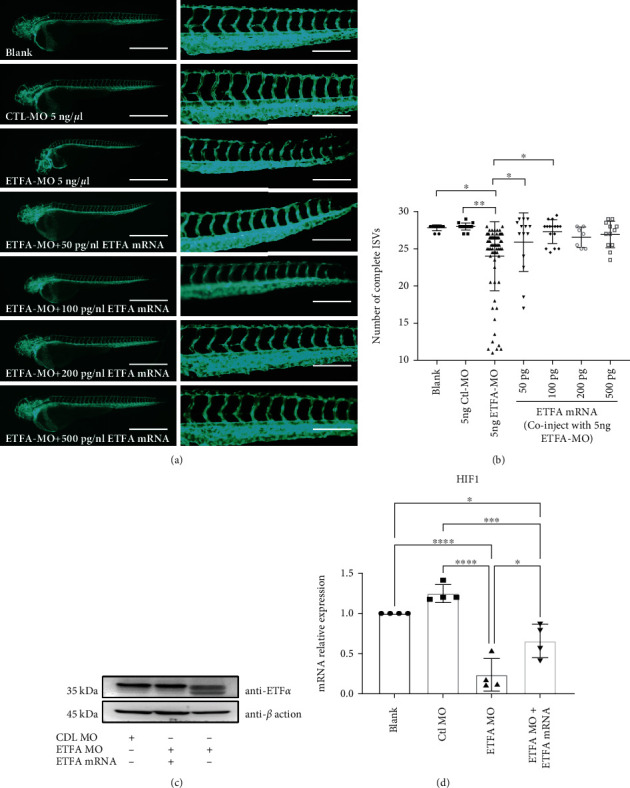
ETF*α* overexpression rescued vascular defects in *ETFA*-MO*-*injected *Tg flK1: EGFP* embryos. *Tg Flk1: EGFP* zebrafish embryos were coinjected 5 ng MO with different concentrations of *ETFA* mRNA as indicated. Embryos were inspected and imaged at 48 hpf. Data presented here were the representative fluorescent images of vascular sprouting (a) and quantitative data of the intact ISVs in different groups (b) (one-way ANNOVA, ^∗^*P* < 0.01, ^∗∗^*P* < 0.001, *n* = 10); (c) representative immunoblot for ETF*α* in zebrafish embryos with the indicated treatments. *β*-Actin was included as the loading control; (d) RT-qPCR analysis of HIF1 gene expression in zebrafish embryos with the indicated treatments. ^∗^*P* < 0.01, ^∗∗∗^*P* < 0.0001, and ^∗∗∗∗^*P* < 0.00001 (one-way ANNOVA, *n* = 4).

An ETFA-specific morpholino oligonucleotide was designed to knockdown ETF*α* expression in zebrafish larvae. This ETFA-specific morpholino specifically binds to its complementary 25-base target RNA sequence at the junction site between intron 2 and exon 3 in the zebrafish ETFA pre-mRNA and interferes in mRNA splicing (Figure S1A). PCR data showed that injection of 5 ng ETFA-MO could significantly reduce ETFA gene expression at both 24 and 48 hours postfertilization (hpf) (Figure S1B). As expected, a much higher mortality was observed in ETFA-MO-injected embryos (Figure 2(a)). Importantly, it was found that ETFA knockdown in live embryos caused a broad spectrum of malformations including delayed development of embryos, defective vessel sprouting, short body, spinal curvature, and curly tail at different time points (Figures 2(b) and 2(c)). Quantitative analysis of these defects showed that ETFA MO-injected embryos exhibited a significant high percentage of defective vessel development (80%) (Figure 2(d)). The defects of vessel development were classified with different severities: incomplete DLAVs (slight), short ISVs (moderate), and inadequate sprouting (severe) at 32 hpf (Figures 3(a) and 3(b)). Accordingly, we found that the number of intact ISVs in the ETFA-MO group was much less than those of the blank control and control MO (Ctrl-MO) groups (Figure 3(c)). Even more severe vessel developmental defects were observed in ETFA-MO-injected embryos at 48 hpf (slight, 45%, moderate, 15%, and severe, 40%) (Figure 4). Importantly, incomplete angiogenic sprouting, which failed to connect with the dorsal longitudinal anastomotic vessel (DLAV) from caudal vessels, was also observed in ETFA-MO-injected embryos (Figure 4(c)). Consistent with this observation, much less intact blood vessels were observed in ETFA-MO-injected embryos, compared with two control groups (Figure 4(e)). These data have collectively demonstrated that ETFA plays a critical role in vessel development. To further testify the function of ETF*α* in vessel development, we replenished ETF*α* protein by injecting exogenous zebrafish ETFA mRNA into ETFA-MO-injected embryos simultaneously. Data shown in Figure 5(c) confirmed that exogenous ETFA mRNA was effectively translated into ETF*α* protein and compensates the loss of ETF*α* in ETFA-MO-injected embryos successively. Consequently, similar numbers of complete ISVs were observed in embryos injected with blank, control-MO, and ETFA-MO plus exogenous ETFA mRNA, while a lower level of intact ISVs was observed in embryos injected with ETFA-MO alone (Figures 5(a) and 5(b)), further confirming the critical role for ETF*α* in vessel sprouting in zebrafish larvae. The expression of the HIF1 gene was also protected by exogenous ETFA mRNA (Figure 5(d)), while knockdown of ETFA could reduce the HIF1 mRNA expression level.

### 3.4. OxLDL Reduces the Protein Level of ETF*α* in Endothelial Cells

**Figure 6:**
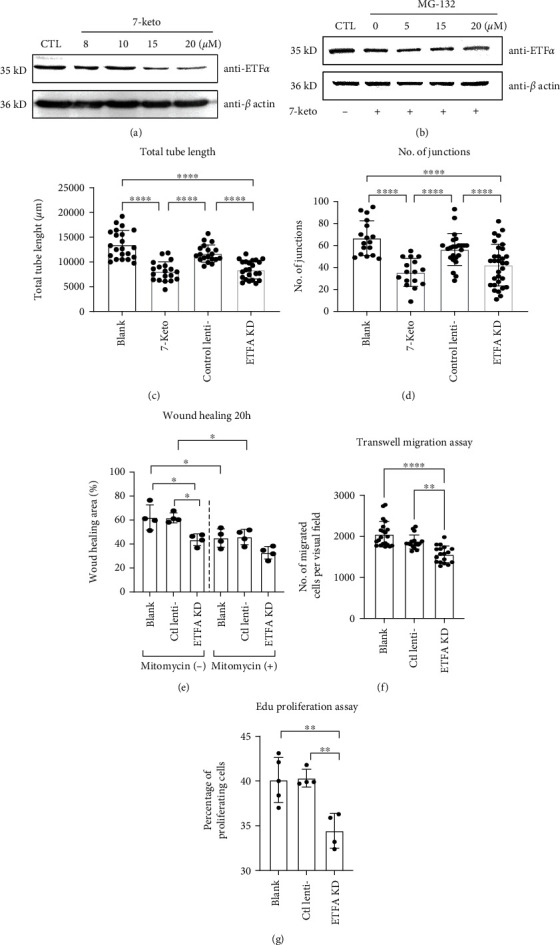
The role of ETF*α* in vessel sprouting in vitro. (a) Representative immunoblot of ETF*α* in HUVECs stimulated by 7-keto cholesterol (8, 10, 15, and 20 *μ*M). GAPDH was used as loading control; (b) representative immunoblot of ETF*α* in HUVECs stimulated by 20 *μ*M 7-keto cholesterol or 7-keto with different doses of MG-132 (5, 15, and 20 nM). *β*-Actin was used as loading control; (c) quantitative analysis of the total tube length and (d) number of junctions in the tube formation assay (one-way ANNOVA, ^∗^*P* < 0.01, ^∗∗^*P* < 0.001, *n* = 20); (e) quantitative analysis of the wound closure area in HUVECs with the indicated treatments at different time points (one-way ANNOVA, ^∗^*P* < 0.01, *n* = 4); (f) quantitative analysis of transwell migration assay in HUVECs with the indicated treatments (one-way ANNOVA, ^∗∗^*P* < 0.001, ^∗∗∗∗^*P* < 0.00001, *n* = 15); (g) quantitative analysis of Edu proliferation assay in HUVECs with the indicated treatments (one-way ANNOVA, ^∗∗^*P* < 0.001, *n* = 4).

7-Keto cholesterol (7-keto) is a bioactive sterol and a major oxysterol component of oxidized low-density lipoprotein (oxLDL). It was found that 7-keto could reduce the protein level of ETF*α* in HUVECs in a dose-dependent manner (Figure 6(a)). Proteasome inhibitor MG-132 had no effect upon decrease of ETF*α* protein expression levels induced by 7-keto treatment, suggesting that the regulation of ETF*α* protein expression by 7-keto is independent of the proteasome pathway. As expected, both 7-keto and ETFA shRNA lentivirus could inhibit EC tube formation significantly with reduced total tube length (sum of branches and segment length per image) and junctions (number of junction per image) (Figures 6(c) and 6(d) and Figure S2B). This result suggests that downregulation of ETF*α* is related to the EC damage induced by oxLDL.

### 3.5. Knockdown of ETF*α* Inhibits Endothelial Migration and Proliferation

The coordination of EC migration and proliferation is essential for both physiological angiogenesis and pathological angiogenesis. Hence, scratch wound assay was utilized to evaluate the importance of ETF*α* in cell proliferation and migration. It was found that knockdown of ETF*α* significantly inhibited wound closure in endothelial cells at 20 hours postscratching when mitomycin C, a cell proliferation inhibitor, was absent (Figure 6(e) and S2). However, the wound healing abilities of control, scramble shRNA-treated, and ETFA knockdown ECs were not significantly different when mitomycin C was present, which suggests that the effect of ETF*α* knockdown on wound healing is less pronounced when cell proliferation is inhibited. To elucidate whether endothelial migration and proliferation are influenced by ETFA knockdown, we carried out transwell migration assay and Edu proliferation assay; the results showed that knockdown of ETF*α* reduces both cell migration and proliferation significantly (Figures 6(f) and 6(g) and Figure S2C, D) but the proliferation was influenced more dramatically.

### 3.6. Knockdown of ETF*α* Reduces the Fatty Acid Oxidation and Oxygen Consumption in HUVECs

**Figure 7:**
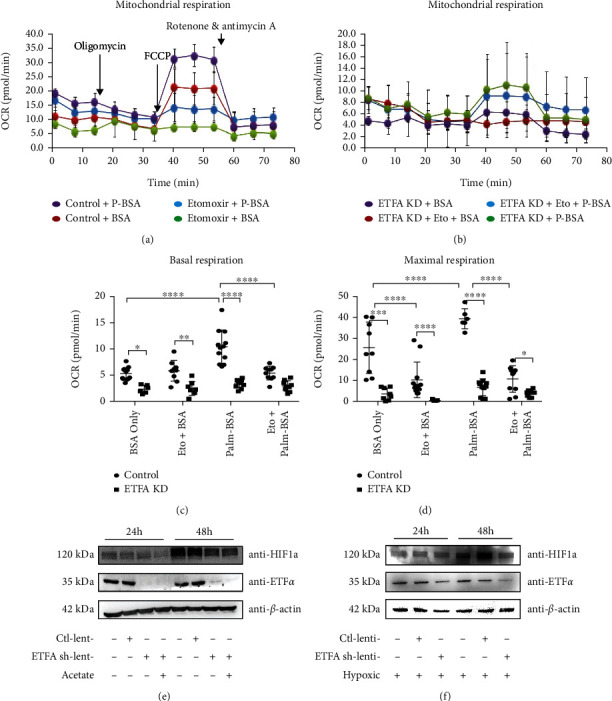
Knockdown of ETF*α* in HUVECs affects mitochondrial bioenergetics and oxygen consumption. (a) Bioenergetic profile of control HUVECs measured by the Seahorse XF analyzer, in which the oxygen consumption rate (OCR) over time is determined. BSA was used to assess utilization of endogenous fatty acid; Palm-BSA was used to assess utilization of exogenous fatty acid. Etomoxir is an inhibitor of fatty acid oxidation (FAO). Oligomycin, an ATP synthase (complex V) inhibitor, was added to detect cellular ATP production; FCCP, a potent uncoupler of oxidative phosphorylation in mitochondria, was added to assess maximal respiration of HUVECS; a mixture of rotenone, a complex I inhibitor, and antimycin A, a complex III inhibitor, was included to measure nonmitochondrial respiration in cells. (b) OCR profiles in ETF*α* knockdown HUVCEs; (c) basal respiration of control and ETF*α* knockdown HUVECs. OCRs in different groups were analyzed with one-way ANNOVA, ^∗∗∗∗^*P* < 0.00001, *n* = 10; (d) maximal respiration of control and ETFA knockdown HUVECs. OCRs in different groups were analyzed with one-way ANNOVA, ^∗∗∗^*P* < 0.0001, ^∗∗∗∗^*P* < 0.00001, *n* = 10; (e) representative immunoblot for HIF1*α* and ETF*α* in control-, scramble shRNA-, and *ETFA* shRNA lentivirus treated HUVECs. Cell lysates were collected at 24 hours or 48 hours after lentivirus transfection. Acetate was added to ETF*α* knockdown HUVECs. *β*-Actin was the loading control; (f) representative immunoblot for HIF1*α* and ETF*α* in control-, scramble shRNA-, and *ETFA* shRNA lentivirus-treated HUVECs. HUVECs were exposed to hypoxic condition for 12 hours before collection. Cell lysates were collected at 24 hours or 48 hours after lentivirus transfection.

It is reported that ECs utilize carbons produced by FAO to fuel biomacromolecule synthesis, which is vital and necessary for cell proliferation [18]. With the combination of XF Palmitate-BSA (Palm-BSA) (FAO substrate), etomoxir (Eto) (an irreversible inhibitor of FAO), and the Seahorse XF Cell Mito Stress Test, the relative utilization of exogenous and endogenous FAs in ECs was determined. In Figure 7(a), Eto plus BSA (green line) and BSA only (red line) were used to evaluate utilization of endogenous FA. Basal respiration (OCR) had no decline in the BSA + Eto group (green) compared with BSA only group (red). This indicates that under quiescent condition, the basal respiration of ECs was due to oxidation of other substrates rather than endogenous FAs. The Palm-BSA + Eto group (blue) was used to determine the oxidation of exogenous FAs. FCCP, a potent uncoupler of oxidative phosphorylation in mitochondria that disrupts ATP synthesis by transporting protons across cell membranes, was used for detecting maximal respiration in ECs. Data shown in Figure 7(a) revealed that compared with control-BAS (red line), a significant increase in maximal respiration was observed in the Palm-BSA group (purple line), while a significant decrease of maximal respiration was observed in Eto + Palm-BSA (blue line) and Eto + BSA (green line) groups. This indicates that exogenous FA (as opposed to endogenous FA) is primarily being oxidized when a bioenergetic stress is applied into ECs. It also suggests that FAs are utilized as a primary energy source under stress. ETF*α* is required for normal mitochondrial FAO and normal amino acid metabolism in the liver [16]; however, its function in EC FAO is still not fully elucidated. We found that knockdown of ETF*α* in ECs significantly reduced the oxygen consumption rates (pmol O_2_/min/well) at baseline from 8–20 pmol O_2_/min/well in control ECs to 4–9 pmol O_2_/min/well in ETFA knockdown ECs (Figures 7(b) and 7(c)). Importantly, no significant increase was observed in terms of maximal respiration after ETFA knockdown (Figures 7(b) and 7(d)). This result suggests that the endothelial cells could not utilize fatty acid under bioenergetic stress when ETF*α* was knockdown, which indicates that ETF*α* plays an important role in FAO of endothelial cells. Additionally, the OCR was lower in ETF*α* knockdown cells than Eto-treated cells, suggesting that ETF*α* is participated in not only oxygen consumption of FAO but also other substrates. These data have collectively suggested that ETF*α* is important for EC FAO and mitochondrial respiration in ECs.

The stability of HIF-1*α* was impaired when ETF*α* was knockdown in endothelial cells.

Hypoxia and the HIF-1*α* pathway are important regulators for EC angiogenesis. HIF-1*α* is stable under hypoxia but degraded under well-oxygenated condition; however, specific metabolic imbalances, or exponentially growing ECs, can accumulate HIF-1*α* in cells, even under normoxic conditions [19]. We have shown that knockdown of ETF*α* could reduce the oxygen consumption rate and EC tube formation, which prompted us to wonder if HIF-1*α* protein expression could be influenced by ETFA knockdown in ECs. Indeed, it was found that HIF-1*α* protein expression was increased in control HUVECs under normal culture condition 48 hours after seeding (Figure 7(e)). However, ETF*α* knockdown significantly reduced HIF-1*α* protein expression. Acetate, which could compensate the lack of acetyl-CoA induced by FAO block in cells, could not increase the HIF-1*α* protein expression when ETFA was knockdown (Figure 7(e)). This result suggests that only compensating the product of FAO could not reverse the degradation of HIF-1*α*, which is mainly regulated by the O_2_ level. It also indicates that defective vessel growth induced by ETF*α* knockdown not only is due to FAO block but also O_2_ consumption related. We also observed that ETF*α* knockdown significantly reduced HIF-1*α* protein expression even under hypoxic condition (Figure 7(f)).

## 4. Discussion

The metabolic switch of endothelial cells is now considered an important driving force of physiological and pathological vascular events [6]. Mitochondria are in the central position of endothelial metabolic homeostasis through supplying energy, signaling, and material needed for biosynthesis [11]. Although mitochondria are not a major ATP source of angiogenic ECs, it still mediates oxidative phosphorylation (OXPHOS) via the electron transport chain (ETC) to produce ATP [5]. The leakage of electrons during ETC is also the major source of cellular ROS, which makes mitochondrion dysfunction important for vascular disease-like atherosclerosis [5, 20]. Our work found that persistent hypercholesterolemia in zebrafish larvae could induce downregulation of several mitochondrion- and metabolic-related enzymes, ETF *α* is one of them, and knockdown of it resulted to defective vessel growth in vivo and vitro. Human ETF are *α*/*β*-heterodimeric FAD-containing proteins in the mitochondrion matrix space [12, 16]. ETF functions as a hub taking up electrons from at least 14 flavoenzymes, feeding them into the respiratory chain [16]. Genetic mutations in the ETFA or ETFB genes cause the Mendelian disorder MADD, which comprises a series of severe symptoms due to energy depletion, impairment of glucose homeostasis and ketogenesis, and the toxic effects of substrates and metabolites [12]. Patient ETF deficiency in human beings is neonatal lethal, and only a fraction of patients with milder signs survives to adulthood [12, 16]. Studies revealed the severity of the symptoms induced by ETF and ETF: QO enzyme deficiencies depend on the residual enzyme activity [12, 16]. It is also proved by our unpublished work that complete deletion of ETFA is neonatal lethal to mice but knockdown of ETFA with morpholino could lead to less than 42% mortality at 48 hpf in zebrafish due to residual ETFA function. However, the surviving larvae had a broad spectrum of deformities, which include vascular malformation. These results suggest an important role of ETF*α*-related mitochondrial function not only in metabolism but also in the vascular homeostasis. ETFA and ETFB are persistently and highly expressed in most tissues, especially in the liver, heart, kidney, skeletal muscle, and fat tissue [12]. Although the ETFA and ETFB are very important for metabolic homeostasis, there is little literature about regulating the mechanism of the two genes' expression. Peroxisome proliferator-activated receptors (PPARs) are a highly potential regulator of ETFA and ETFB according to the bioinformatic prediction, and the former one is also an important regulator of fatty acid oxidation enzymes [12]. Our group will also focus on the relationship between PPARs and ETFA during angiogenesis next. Given the stable expression of ETF, its activity might be regulated by posttranslational modifications (PTMs). Our preliminary proteomics data and others' publication showed both that ETF-*α* and ETF-*β* subunits possess abundant PTMs, which comprises carbonylation, acetylation, glutarylation or succinylation, phosphorylation, and trimethylation [12]. However, the exact meaning and function of these PTM for ETF's activity are not clear and need further investigation.

The vascular system is a highly branched network that supplies surrounding tissues with oxygen (O_2_) and nutrients [12, 15, 16]. Formation of the vascular network occurs from embryogenesis to adulthood and is stimulated by the oxygen and metabolism demands of organ growth and function [9]. The hypoxic tissues produce proangiogenic stimuli and initiate migration and proliferation of ECs in preexisting vessels to establish new vessels [16–20]. During this process, the migrating ECs utilize glycolysis to produce ATP and the proliferating ECs use FAO to supply the carbons needed for dNTP synthesis [10, 24, 25]. Once nutrient and O_2_ supplies meet tissue demands, the sprouting process is terminated and ECs become quiescent [25]. ECs sense O_2_ tension with many sensors, which include NADPH oxidases, endothelial nitric oxide synthase (eNOS), heme oxygenases, mitochondria, and 2-oxoglutarate-dependent iron(ii) dioxygenase superfamily [25]. These sensors interface with the hypoxia-inducible transcription factor (HIF) family, which includes three isoforms HIF*α* (HIF-1–3) [26]. All three isoforms of HIF*α* can heterodimerize with the HIF*β*/ARNT subunit to form an active transcriptional complex that initiates expression of hundreds of angiogenic genes [25]. The activity of these HIFs is regulated by the O_2_ sensors, including prolyl hydroxylase domain proteins (PHD1–3) and factor inhibiting HIFs (FIH), all of which belong to the 2-oxoglutarate-dependent iron (ii) dioxygenase superfamily [27]. During normoxia, PHDs use O_2_ to hydroxylate specific proline residues of HIF proteins, which lead to degradation through the von Hippel-Lindau (VHL) E3 ubiquitin ligase complex-regulated proteasomal pathway [27]. Under hypoxia, HIFs become stable due to the reduction of PHD/FIH activity, triggering HIF's target gene expression, which subsequently regulate cell metabolism, proliferation, and angiogenesis [26, 27]. However, there are exceptions. Such specific metabolic imbalances, or the cell culture density, can induce HIF1*α* accumulation, even under normoxic conditions through a not well-understood mechanism [19, 28]. It is confirmed by our work that ETF*α* takes part in FAO; knockdown of which would influence endothelial proliferation more than migration. It is consistent with the statement that endothelial cell relies on glycolysis to supply energy when migrating, while needing FAO to provide materials for biosynthesis. Knockdown of ETF*α* would also reduce the protein level of endothelial HIF1*α*, which is in consistency with impaired vessel sprouting in ETFA MO-injected zebrafish larvae. The underlying mechanism behind it is probably related to O_2_ consumption decrease in ETFA knockdown endothelial cells.

Taken together, we demonstrate that downregulation of ETF*α* is involved in hypercholesterolemia-induced vascular malfunctions in zebrafish larvae. Our data show that ETF*α* plays an important role in vessel development and angiogenesis by modulating mitochondrial FAO and HIF1*α* expression levels in ECs. However, the molecular mechanism underling this process needs further investigation. Our results link endothelial function regulated by ETF*α* with impaired angiogenesis during pathological conditions like hypercholesterolemia. These findings may shed some light on the mechanism of endothelial dysfunction that precedes the formation of atherogenic plaques, as well as for the cause of impaired collateral vessel growth that is observed in patients with hypercholesterolemia [29].

## Figures and Tables

**Table 1:** Downregulated or upregulated mitochondrion-related proteins in high-cholesterol diet-fed zebrafish larvae identified by isobaric tags for relative and absolute quantitation (iTRAQ) analysis.

Accession no.	Gene	Protein	Human ortholog	Fold change (control-vs-HCD)	Significance
ENSDARP00000022528	atp5f1d	ATP synthase F1 subunit delta	ATP5F1D (ATP synthase F1 subunit delta)	1.608	^∗^
ENSDARP00000041699	prdx3	Peroxiredoxin 3	PRDX3 (peroxiredoxin 3)	1.481	^∗^
ENSDARP00000067447	acat1	Acetyl-CoA acetyltransferase 1	ACAT1 (acetyl-CoA acetyltransferase 1)	1.309	^∗^
ENSDARP00000064522	etfa	Electron transfer flavoprotein subunit alpha	ETFA (electron transfer flavoprotein subunit alpha)	1.231	^∗^
ENSDARP00000055709	aldh4a1	Aldehyde dehydrogenase 4 family, member A1	ALDH4A1 (aldehyde dehydrogenase 4 family member A1)	1.219	^∗^
ENSDARP00000028117	aco2	Aconitase 2, mitochondrial	ACO2 (aconitase 2)	1.216	^∗^
ENSDARP00000007446	cox4i1	Cytochrome c oxidase subunit 4I1	COX4I1 (cytochrome c oxidase subunit 4I1)	1.205	^∗^
ENSDARP00000076104	prdx1	Peroxiredoxin 1	Peroxiredoxin 1	0.832	^∗^
ENSDARP00000092919	cox17	COX17 cytochrome c oxidase assembly homolog	COX17 cytochrome c oxidase assembly homolog	0.828	^∗^
ENSDARP00000055160	cox6b2 zgc:66195	Cytochrome c oxidase subunit	Cytochrome c oxidase subunit	0.765	^∗^
ENSDARP00000090664	atp5meb	ATP synthase subunit e, mitochondrial	ATP synthase, H+-transporting, mitochondrial Fo complex, subunit Eb	0.745	^∗^

## Data Availability

The data could be found in our manuscript and supplementary data.

## Abbreviation

CVD: Cardiovascular disease
Dpf: Days post fertilization
DA: Dorsal aorta
DLAV: Dorsal longitudinal anastomotic vessel
DS: Defective sprouting
EC: Endothelial cells
eNOS: Endothelial nitric oxide synthase
ETF-*α*: Electron transfer flavoprotein-*α*
Eto: Etomoxir
ETC: Electron transport chain
FAO: Fatty acid oxidation
HIF1*α*: Hypoxia-inducible factor-1*α*
HCD: High-cholesterol diet
Hpf: Hours postfertilization
ISVs: Intersegmental vessels
MADD: Multiple acyl-CoA dehydrogenase deficiency
MO: Morpholino antisense oligonucleotides
oxLDL: Oxidized low-density lipoprotein
PPARs: Peroxisome proliferator-activated receptors
PTMs: Posttranslational modifications
7-keto: 7-Keto cholesterol.

## Supplementary Materials

Supplementary 1 Supplementary Figure 1: (A) design of morpholino towards the *ETFA* gene in zebra fish larvae. The *ETFA*-MO was targeted towards the junction site between intron region 2 and exon 3 which leads to the disturbance of *ETFA* mRNA; (B) representative RT-qPCR result for the knockdown effects of *ETFA*-MO in *Tg flk1: EGFP* zebrafish larvae at 24 and 48 hours past fertilization. Supplementary Figure 2: (A–F) RT-qPCR analysis of downregulated genes screened out by iTRAQ in HCD-fed zebrafish larvae. ^∗^*P* < 0.01 (one-way ANNOVA, *n* = 3). Supplementary Figure 3: (A) representative immunoblot for ETF*α* in HUVECs with the indicated treatments. *β*-Actin was included as the loading control; (B) representative images showing tube formation of HUVECs stimulated by 7-keto, infected with scramble shRNA or *ETFA* shRNA lentivirus; (C) representative images illustrating scratch wound healing in MitoC-treated control and ETF*α* knockdown HUVECs at different time points; (D) representative images of transwell migration assay; (E) representative images of Edu proliferation assay with flow cytometry. Supplementary Figure 4: (A) quantitative data for HIF1*α* relative expression levels in Figure 5(e); (B) quantitative data for HIF1*α* relative expression levels in Figure 5(f).

Supplementary 2 Supplementary data 2. Original images of Western blot data included in respective figures.
