# Supplementary figures and images for: Electron Transfer Flavoprotein (ETF) α Controls Blood Vessel Development by Regulating Endothelial Mitochondrial Bioenergetics and Oxygen Consumption
Source: Oxid Med Cell Longev. 2022 Mar 11;2022:7969916. doi: 10.1155/2022/7969916 (PMC8933654; doi:10.1155/2022/7969916)

**A**

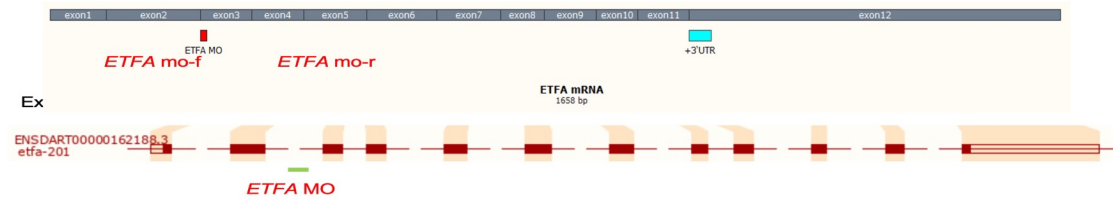

**B**

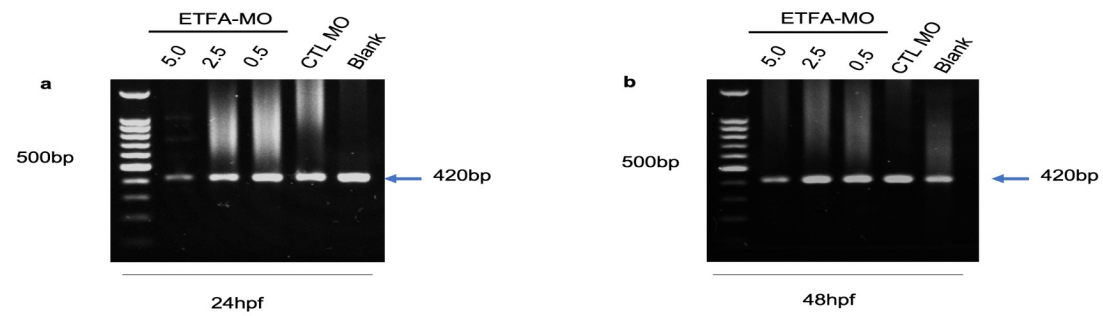

**Figure S1**

**A**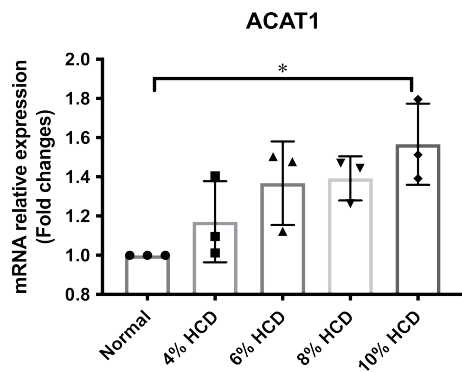**B**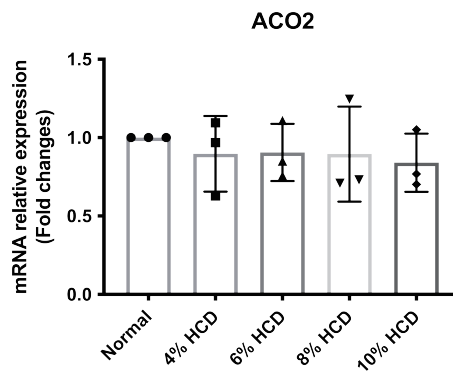**C**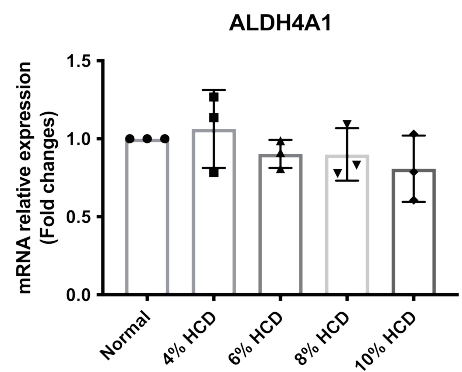**D**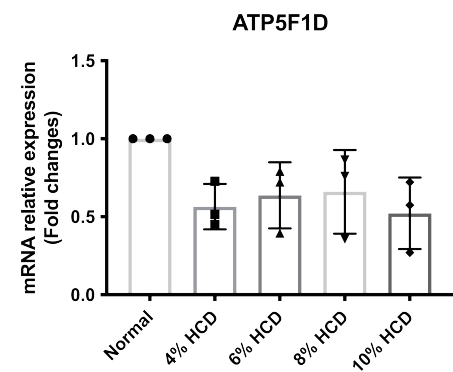**E**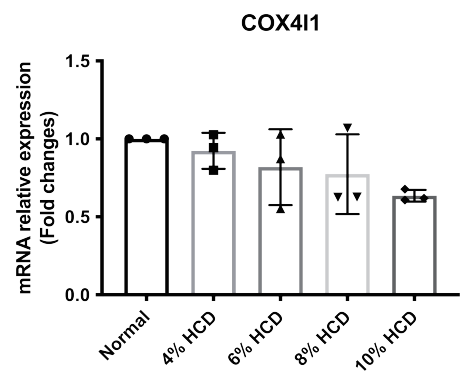**F**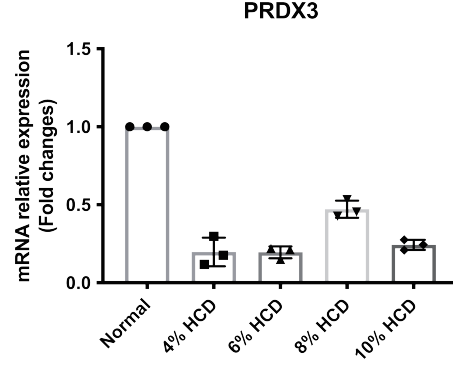**Fig. S2**

A

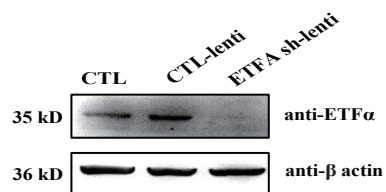

B

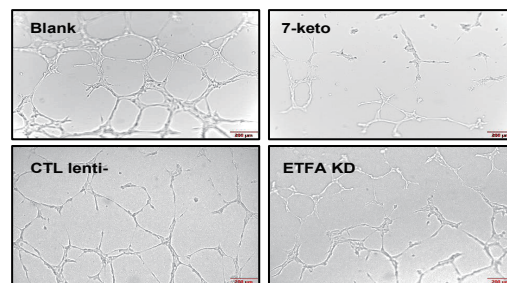

C

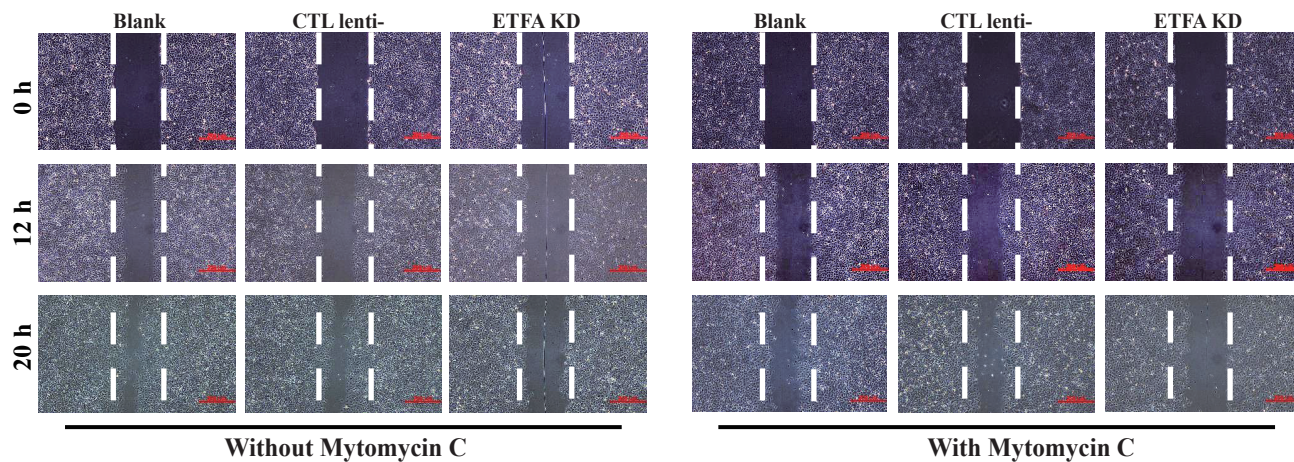

D

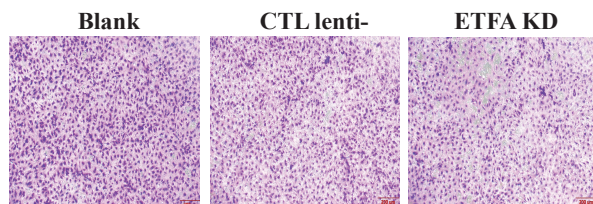

E

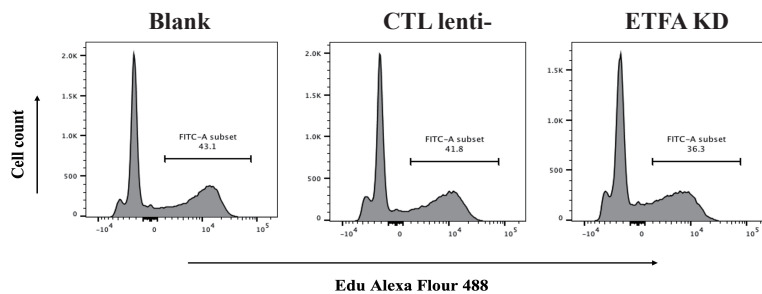

Fig. S3

A

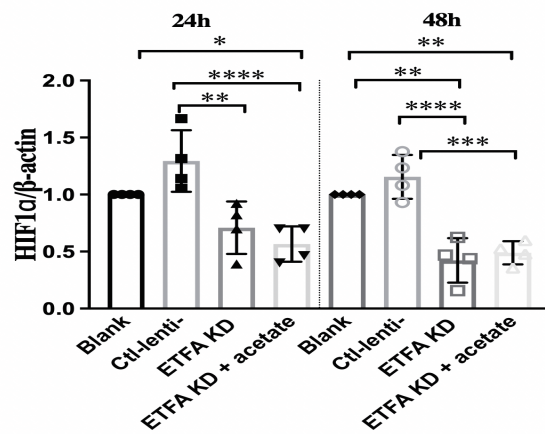

B

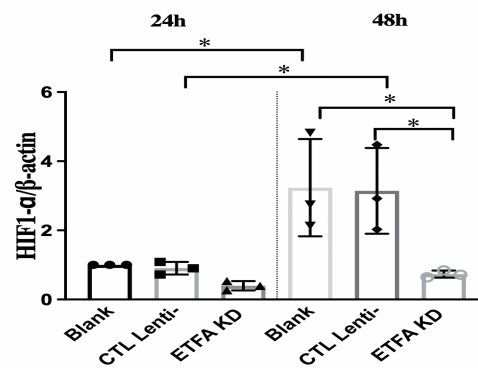

Fig. S4

Supplement: Supplementary 1 — Supplementary Figure 1: (A) design of morpholino towards the ETFA gene in zebra fish larvae. The ETFA-MO was targeted towards the junction site between intron region 2 and exon 3 which leads to the disturbance of ETFA mRNA; (B) representative RT-qPCR result for the knockdown effects of ETFA-MO in Tg flk1: EGFP zebrafish larvae at 24 and 48 hours past fertilization. Supplementary Figure 2: (A–F) RT-qPCR analysis of downregulated genes screened out by iTRAQ in HCD-fed zebrafish larvae. ∗P < 0.01 (one-way ANNOVA, n = 3). Supplementary Figure 3: (A) representative immunoblot for ETFα in HUVECs with the indicated treatments. β-Actin was included as the loading control; (B) representative images showing tube formation of HUVECs stimulated by 7-keto, infected with scramble shRNA or ETFA shRNA lentivirus; (C) representative images illustrating scratch wound healing in MitoC-treated control and ETFα knockdown HUVECs at different time points; (D) representative images of transwell migration assay; (E) representative images of Edu proliferation assay with flow cytometry. Supplementary Figure 4: (A) quantitative data for HIF1α relative expression levels in Figure 5(e); (B) quantitative data for HIF1α relative expression levels in Figure 5(f). [file 7969916.f1.pdf]
