# Supplementary material for: Electron Transfer Flavoprotein (ETF) α Controls Blood Vessel Development by Regulating Endothelial Mitochondrial Bioenergetics and Oxygen Consumption
Source: Oxid Med Cell Longev. 2022 Mar 11;2022:7969916. doi: 10.1155/2022/7969916 (PMC8933654; doi:10.1155/2022/7969916)

Original data of Fig. 5C

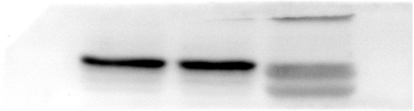

ETFA

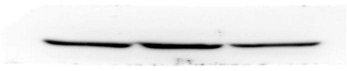

β-actin

Original data of Fig. 6A

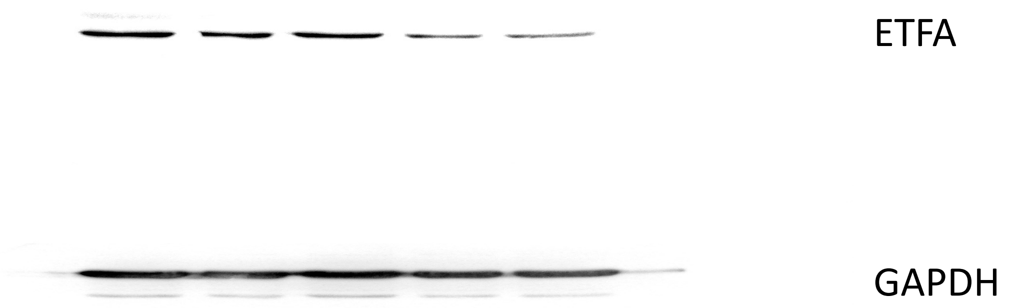

Original data of Fig. 6B

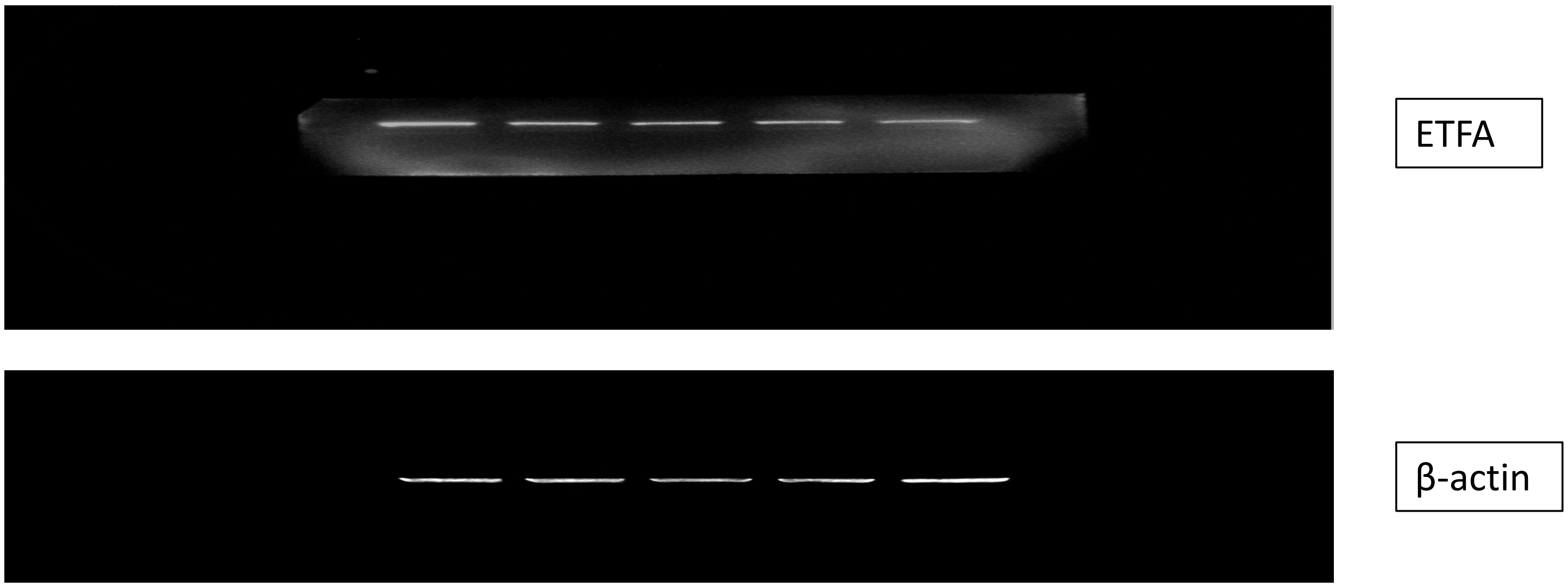

Original data of Fig. 7E

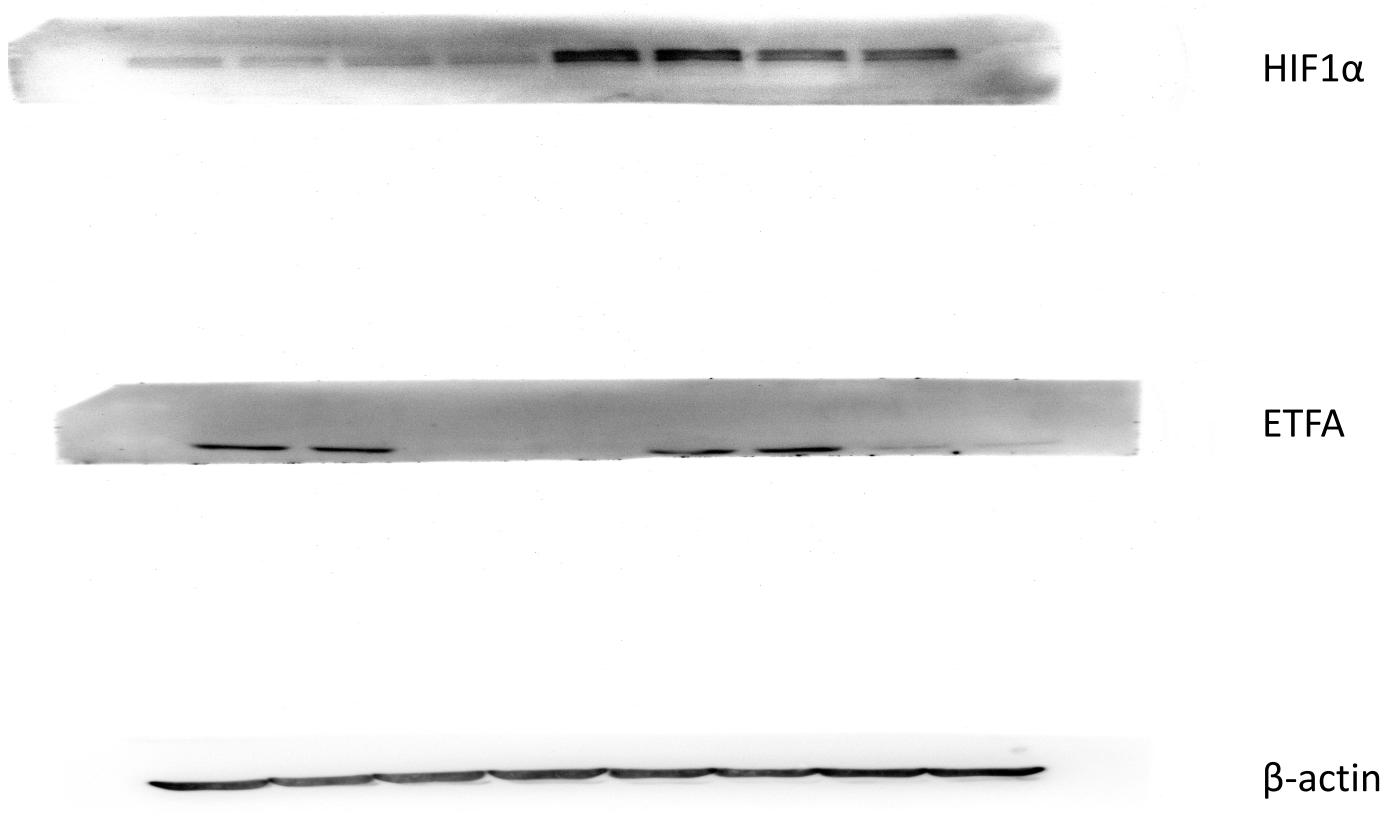

Original data of Fig. 7G

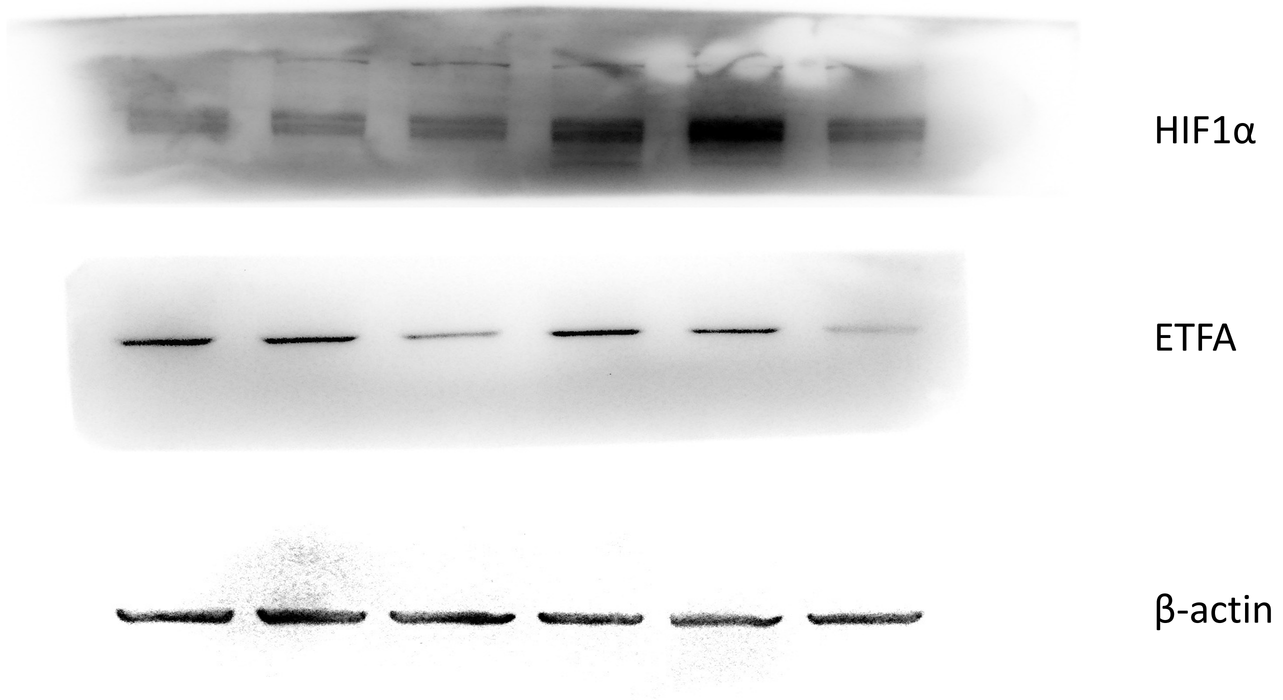

Supplement: Supplementary 2 — Supplementary data 2. Original images of Western blot data included in respective figures. [file 7969916.f2.pdf]
